# Publisher Correction: Charging dynamics of an individual nanopore

**DOI:** 10.1038/s41467-019-09701-0

**Published:** 2019-05-02

**Authors:** Ran Tivony, Sam Safran, Philip Pincus, Gilad Silbert, Jacob Klein

**Affiliations:** 1 Department of Materials and Interfaces, Weizmann Institute of Science, Rehovot, 76100 Israel; 2 Physics Department, University of California, Santa Barbara, CA 93106 USA; 3 Present Address: Adama Makhteshim Ltd., Beer Sheva, 84100 Israel

**Keywords:** Physical chemistry, Materials for energy and catalysis

Correction to: *Nature Communications* 10.1038/s41467-018-06364-1, published online 5 Feb 2019.

The original version of this Article contained an error throughout in which an incorrect symbol was used for the diffusion coefficient: it should be cambria math, italicized, and not bold. These have been corrected in both the PDF and HTML versions of the Article.

