# Supplementary material for: Charging dynamics of an individual nanopore
Source: Nat Commun. 2018 Oct 11;9:4203. doi: 10.1038/s41467-018-06364-1 (PMC6181992; doi:10.1038/s41467-018-06364-1)
Supplement: Supplementary file 1 — Supplementary information [file 41467_2018_6364_MOESM1_ESM.pdf]

Supplementary information

Charging dynamics of an individual nanopore

Tivony et al.

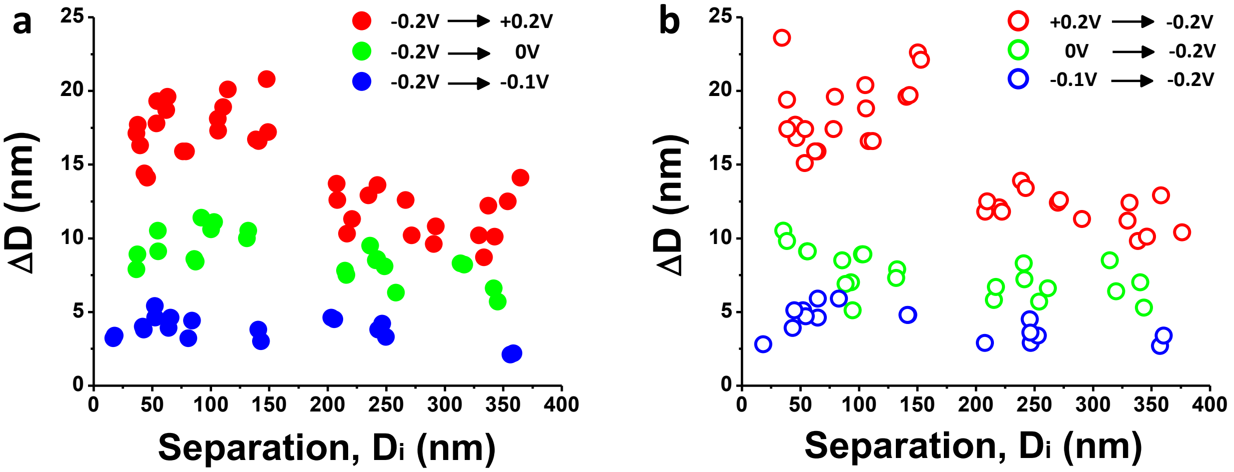

Supplementary Figure 1. Separation shift ΔD measurement at various surface distances D_i following an application of different positive potential steps **(a)** and negative potential steps **(b)**, as indicated in figure legends.

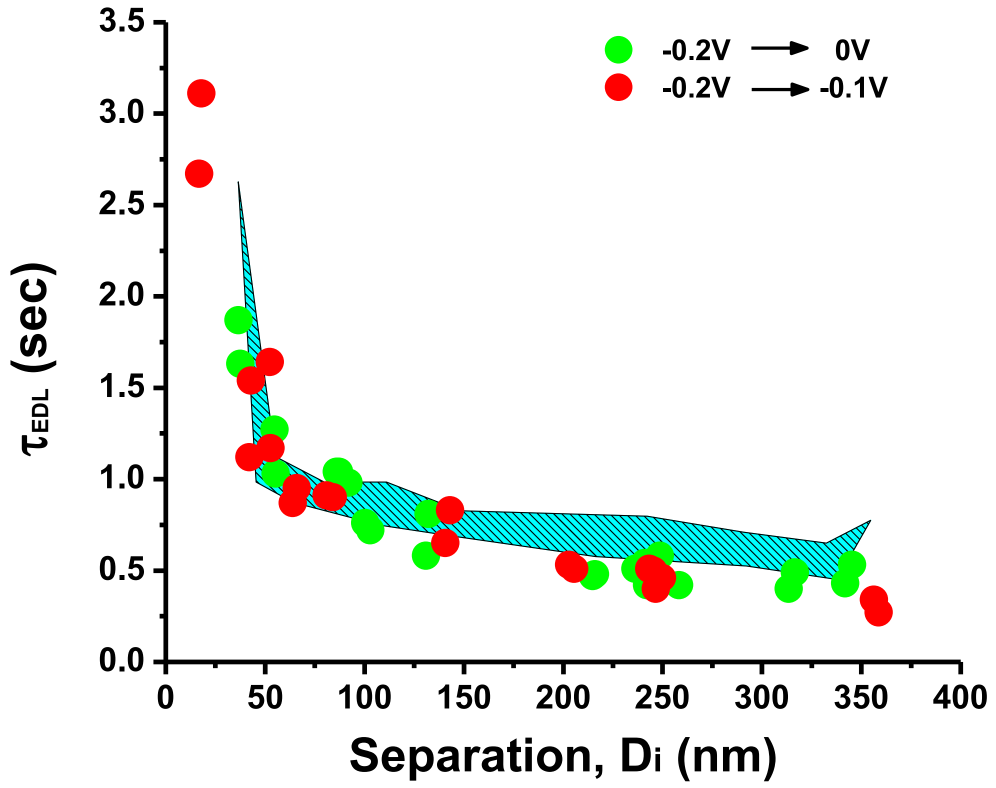

Supplementary Figure 2. Measured EDL charging time τ_{EDL} as a function of surface separations (D_i) and at different applied potentials, as indicated in legend. The shaded area, presented for comparison, corresponds to τ_{EDL} measurements at potential step of −0.2V → +0.2V, also depicted in Figure 4b (green symbols). All measurements were taken in 2mM $NaNO_3$ solution.

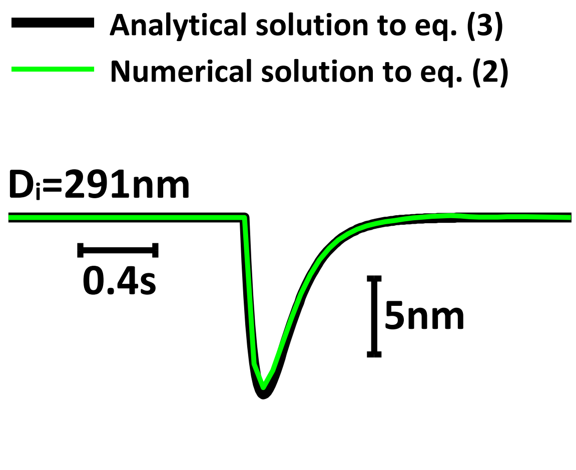

Supplementary Figure 3. Comparison between approximate analytical solution (Supplementary Equation (2)) and exact numerical solution of Supplementary Equation (1) (Equation (3) in the main text), for two values of D_i spanning the range in our experiments. Parameters used are fully determined and are as follows: $\Psi_{gold,eff}$ = 0.105 V (determined as detailed in section (1) above, Ψ_{mica} = −0.05 V, η_{eff} = 8.9 × 10^{-4} Pa s, R = 0.01 m, K_n = 80 N m^{-1}, and ε = 80. The characteristic τ value for each trace was calculated separately using equation (2') in the main text at t = Δt_s (obtained from the experimental peaks), τ = 0.149 s and 0.165 s for Di = 291 nm and 112 nm, respectively.

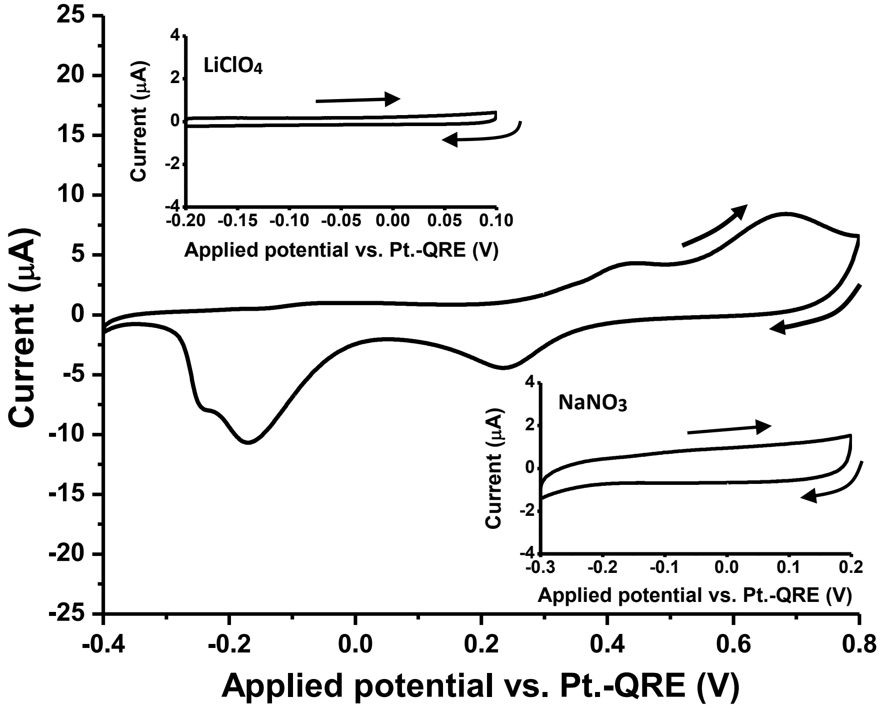

Supplementary Figure 4. Cyclic voltammetry (CV) measurement of a molecularly-smooth gold electrode in 2mM $LiCiO_4$ in the range between -0.4 and 0.8V showing its characteristic oxidation and reduction peaks. Inset: CV measurements in 2mM $LiClO_4$ in the range between −0.2 to 0.1V (top) and in 1mM $NaNO_3$ in the range between −0.3 to 0.2V (bottom). Both measurements were taken in the so-called electric double-layer region, where the electrode is ideally polarized, indicating that neither oxidation not reduction occur at the gold surface. CV measurements in $LiClO_4$ were taken using the three-electrode configuration in the SFB with the gold as a working electrode and platinum both as a counter and as a (quasi) reference electrode. Measurements in $NaNO_3$ were taken using a similar three-electrode configuration using an external electrochemical cell.

Supplementary note 1: Estimate of effective gold surface potential

The potential applied at the gold surface at t = 0 by $\Delta\Psi_{app}$, changes the equilibrium potential at the gold surface from Ψ_{gold1} to Ψ_{gold2}; these equilibrium values can extracted from the normal force profiles (Figure 2 in main text). However, the instantaneous effective potential at the gold surface at t = 0 must depend both on Ψ_{gold1} and on Ψ_{gold2}. The reason for this is as follows: At t < 0 the ion cloud in the vicinity of the gold surface exactly neutralizes the charge on the gold which is at a surface potential Ψ_{gold1}. At t = 0 the gold surface charge instantaneously changes but the ion cloud has not yet had time to adjust to its equilibrium value (which happens only after a time τ_{EDL}). Thus the effective potential Ψ_{eff} at the gold surface at t = 0 arises from that due to the 'old' charge cloud (t < 0) in the vicinity of the gold surface, together with that due to the new charge on the gold surface induced by $\Delta\Psi_{app}$. This is consistent with the shift ΔD in separation between the surfaces on application of $\Delta\Psi_{app}$, as shown in Supplementary Figure 1.

We see in Supplementary Figure 1 that the force between the surfaces ($F = k_n\Delta D$) is the same whether they go from Ψ_{gold1} to Ψ_{gold2} or from Ψ_{gold2} to Ψ_{gold1}. This demonstrates that the force — and thus the effective potential at t = 0 — does not depend on either Ψ_{gold1} or Ψ_{gold2} by themselves, but on their combination. Since the potential at the surface arises in part from the 'old' ion charge cloud associated with Ψ_{gold1} and in part from the new surface charge associated with Ψ_{gold2}, we may estimate the effective surface potential instantaneously following $\Delta\Psi_{app}$ at t = 0, as $\Psi_{eff} = (\Psi_{gold1} - \Psi_{gold2})/2$. This is the value used in the expression for F_e in eq. (2) of the main text.

Supplementary note 2: Estimate of effective radius L of confined nano-pore within the transmission line model

The transmission line model for charging within a cylindrical nano-pore[1] considers a cylinder of length L and thickness h_p, closed at one end and exposed to the bulk solution (i.e. a reservoir of ions) at its open end, so that L is the distance that ions from the reservoir need to diffuse to lead to EDL charging across the entire nanopore. Our present configuration (Figure 2b in main text) is a disk-like pore symmetric about its mid-plane, confined between two orthogonally-crossed cylinders, mean radius R (≈ 1 cm) and closest separation D_i (≈ O(100 nm)). The effective geometry of such a configuration both for electrostatic[2] and hydrodynamic[3] interactions is that of a sphere (radius R) on a flat a closest distance D_i apart (in the Derjaguin approximation[2], which applies well for the present case of $D_i << R$). The thickness of the disk varies between D_i and $(D_i + (r^2/2R))$ at a distance r from the center.

We look for an effective diameter r corresponding to the length L of the cylinder in the TL model. Thus in our configuration L is the distance that ions from a region of sufficient ion excess (corresponding to the ion reservoir in the TL model) need to diffuse to the disk center. Consider a change in the potential of the gold which results in a change $\Delta\sigma$ in the surface charge density. This will lead to a change $\Delta\sigma$/unit area of ions that need to be compensated. We assume that ions need to travel to the disk center (r = 0) from a region at r = L where the excess number of ions in the gap relative to that at the disk center exceeds the number needing to be replaced. At r = L the excess gap thickness δ relative to the center (r = 0) is $\delta = (L^2/2R)$, and the excess of ions relative to the disk center is then δc_0/unit area where c_0 is the bulk ion concentration (number of ions per unit volume). We thus require $\delta c_0 e > \sigma$, or $L > (2R\Delta\sigma/c_0 e)^{1/2}$, where e is the electronic charge (assuming singly-charged

ions). This is roughly the distance that ions need to travel to the disk center from a region (r = L) where there is sufficient ion excess to compensate for the charge density change at the disk center (r = 0) arising from the applied potential change $\Delta\Psi_{app}$, so that it can act as a reservoir.

To be specific, we take the potential change $\Delta\Psi_{app}$: +0.2V → –0.2V in the 2mM $NaNO_3$ solution. From Figure 2a in main text, this corresponds to a change in gold surface potential from +0.04V to −0.17V. From the Grahame equation, $\sigma = (8c_0\varepsilon\varepsilon_0 kT)^{1/2}\sinh(ze\Psi_{gold}/2kT)$, and putting in values (Ψ_{gold} = −0.17V, and Ψ_{gold} = +0.04V) we find $\Delta\sigma = 72 \times 10^{-3}$ C m^{-2}, so that $L > (2R\Delta\sigma/c_0 e)^{1/2} \approx 87$ μm.

Supplementary note 3: EDL charging dynamics at different applied potentials

The surface charge density difference of the gold for the two potential steps shown in Supplementary Figure 2 (−0.2V → −0.1V, and −0.2V → 0V) may be evaluated as indicated in section 2 above and yield $\Delta\sigma = 69.4 \times 10^{-3}$ C m^{-2}and 69.5 x 10^{-3} C m^{-2}, respectively. These values compare with $\Delta\sigma = 72 \times 10^{-3}$ C m^{-2} for the −0.2V → +0.2V potential changes corresponding to the shaded band in Supplementary Figure 2. Since $L \gtrsim (2R\Delta\sigma/c_0 e)^{1/2}$ and the characteristic time $\tau_{TL} = (L^2\lambda_D/\mathcal{D}h_p)$, this suggests that the EDL charging time for the potential steps in Supplementary Figure 2 (circles) should be some 4–5% shorter compared with the data in the shaded band, which is indeed roughly the case.

Supplementary note 4: Numerical solution of dynamical equation

We solved numerically (Wolfram Mathematica) equation (2) in the main text:

$[\pi R\varepsilon\varepsilon_0(\Delta\Psi_{eff})^2/D(t)]e^{-t/\tau} = K_n\Delta D(t)+ 6\pi R^2\eta[(dD/dt)/D(t)]$ (Supplementary Equation 1)

with boundary condition $D = D_i$ at $t = 0$, and compared our results with the prediction of the approximate solution given in equation(3) of the main text:

$D(t) = D_i + [F_e(0)\tau e^{-t/\tau} (\exp(t(f_H - K_n\tau)/f_H\tau) - 1)]/(K_n\tau - f_H)$ (Supplementary Equation 2)

where $F_e(0) = \pi R\varepsilon\varepsilon_0(\Delta\Psi_{eff})^2/D_i$ and $f_H = 6\pi R^2\eta/D_i$. The comparison is shown in Supplementary Figure 3, and reveals a close fit at larger D_i values, with a good but less close fit at lower D_i; this is as expected as the approximation leading to equation (3) in the text (Supplementary Equation(2) above) is best at higher D_i values.

Supplementary note 5: Cyclic voltammetry of gold

We carried out cyclic voltammetry on our gold surfaces to verify no chemical reactions were occurring (ideal or faradaic regime) at the potentials used in our experiments, as in Supplementary Figure 4.

Supplementary note 6: Parameters used for dynamic equations

The parameters used in equation(3) in main text for plotting the peaks in Figure 3b are all fully determined either from the force distance profiles (for the case of the potentials) or from the peak position in the D(t) traces, for the case of τ, and are as follows: $\Psi_{gold,eff}$ = 0.105 V (determined as detailed in section (1) above, $\Psi_{mica} = -0.05$ V, $\eta_{eff} = 8.9 \times 10^{-4}$ Pa.s, R = 0.01 m, $K_n = 80$ N m^{-1}, and $\varepsilon = 80$. The characteristic time τ for each trace was calculated separately using equation (2') at $t = \Delta t_s$, yielding t = 0.149 s, 0.156 s and 0.165s for D_i = 291 nm, 209 nm and 112 nm respectively.

Parameters used to generate curves in Figure 4: Solid and dotted black curves in Figure 4a and upper black curve in Figure 4b, all in 2mM $NaNO_3$, are from equation(3) with the same parameters as for Figure 3b above. Lower black curve in Figure 4b is from a different experiment, in 5 mM NaNO3, for which the parameters were determined separately: $\Psi_{gold,eff}$ = 0.084 V (determined as detailed in section (1) above, Ψ_{mica} = −0.085 V, τ = 0.03 s, η_{eff} = 8.9 × 10^{-4} Pa s, R = 0.01 m, ε = 80 and K_n = 156 N m^{-1} (we note the different measured spring constant for this experiment). No adjustable parameters are used in any of the curves.

Supplementary References

1. De Levie, R. On porous electrodes in electrolyte solutions: I. Capacitance effects. *Electrochim. Acta* **8**, 751–780 (1963).
2. Israelachvili, J. N. *Intermolecular and Surface Forces*. 2nd edn, (Academic Press Limited, 1992).
3. Klein, J. *et al.* Lubrication Forces between Surfaces Bearing Polymer Brushes. *Macromolecules* **26**, 5552–5560 (1993).
